# *N*,*N*′-Dibenzyl-*N*′′-(2,6-difluoro­benzo­yl)-*N*,*N*′-dimethyl­phospho­ric triamide

**DOI:** 10.1107/S1600536810035725

**Published:** 2010-09-11

**Authors:** Mehrdad Pourayoubi, Atekeh Tarahhomi, Arnold L. Rheingold, James A. Golen

**Affiliations:** a Department of Chemistry, Ferdowsi University of Mashhad, Mashhad 91779, Iran; b Department of Chemistry, University of California, San Diego, 9500 Gilman Drive, La Jolla, CA 92093, USA

## Abstract

The phosphoryl and carbonyl groups in the title compound, C_23_H_24_F_2_N_3_O_2_P, are *anti* to each other. The P atom is in a tetra­hedral coordination environment and the environment of each N atom is essentially planar, the average bond angles at the two N atoms being 119.9 and 119.1°. The H atom of the C(=O)NHP(=O) group is involved in an inter­molecular –P=O⋯H–N– hydrogen bond, forming centrosymmetric dimers.

## Related literature

For related structures, see: Pourayoubi & Sabbaghi (2009 ▶); Sabbaghi *et al.* (2010 ▶).
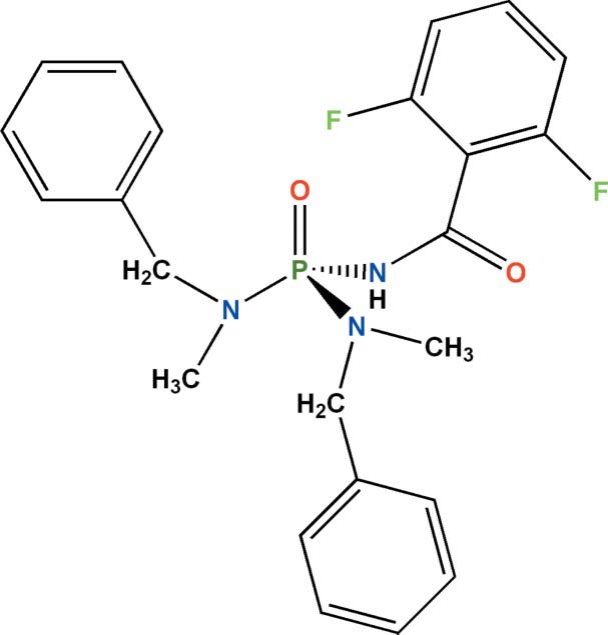


## Experimental

### 

#### Crystal data

C_23_H_24_F_2_N_3_O_2_P
*M* *_r_* = 443.42
Triclinic, 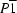

*a* = 9.9370 (11) Å
*b* = 11.1093 (15) Å
*c* = 11.5902 (14) Å
α = 89.101 (4)°
β = 67.826 (4)°
γ = 71.664 (4)°
*V* = 1116.9 (2) Å^3^
*Z* = 2
Mo *K*α radiation
μ = 0.16 mm^−1^
*T* = 200 K
0.30 × 0.25 × 0.20 mm

#### Data collection

Bruker SMART X2S benchtop CCD area-detector diffractometer
Absorption correction: multi-scan *SADABS* (Bruker, 2005 ▶) *T*_min_ = 0.952, *T*_max_ = 0.968
13602 measured reflections
5173 independent reflections
3851 reflections with *I* > 2σ(*I*)
*R*_int_ = 0.036

#### Refinement

*R*[*F* ^2^ > 2σ(*F* ^2^)] = 0.048
*wR*(*F* ^2^) = 0.126
*S* = 1.03
5173 reflections
286 parameters
H atoms treated by a mixture of independent and constrained refinement
Δρ_max_ = 0.37 e Å^−3^
Δρ_min_ = −0.32 e Å^−3^

### 

Data collection: *SMART* (Bruker, 2005 ▶); cell refinement: *SAINT* (Bruker, 2005 ▶); data reduction: *SAINT*; program(s) used to solve structure: *SHELXS97* (Sheldrick, 2008 ▶); program(s) used to refine structure: *SHELXL97* (Sheldrick, 2008 ▶); molecular graphics: *SHELXTL* (Sheldrick, 2008 ▶); software used to prepare material for publication: *SHELXTL*.

## Supplementary Material

Crystal structure: contains datablocks I, global. DOI: 10.1107/S1600536810035725/ng5027sup1.cif

Structure factors: contains datablocks I. DOI: 10.1107/S1600536810035725/ng5027Isup2.hkl

Additional supplementary materials:  crystallographic information; 3D view; checkCIF report

## Figures and Tables

**Table 1:** Hydrogen-bond geometry (Å, °)

*D*—H⋯*A*	*D*—H	H⋯*A*	*D*⋯*A*	*D*—H⋯*A*
N1—H1*A*⋯O2^i^	0.83 (2)	1.92 (2)	2.752 (2)	173 (2)

Symmetry code: (i) 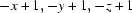.

**Fig. 1.**
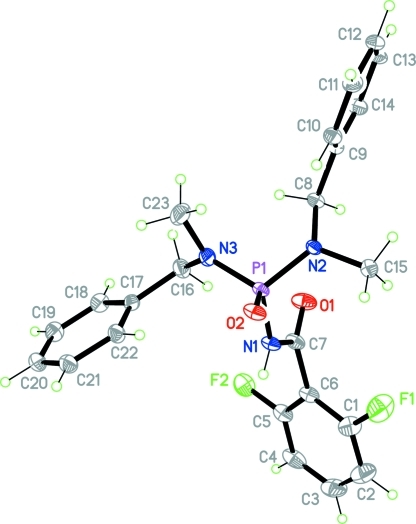
An ORTEP-style plot of title compound. Ellipsoids are given at the 50% probability level.

## supplementary crystallographic information

### Comment

Following the previous works about carbacylamidophosphates with a C(═O)NHP(═O) skeleton such as P(O)[NHC(O)C_6_H_4_(4-NO_2_)][N(CH(CH_3_)_2_)(CH_2_C_6_H_5_)]_2_ (Pourayoubi & Sabbaghi, 2009) and P(O)[NHC(O)C_6_H_4_(4-NO_2_)][NHC_6_H_11_]_2_ (Sabbaghi *et al.*, 2010), here, we report the synthesis and crystal structure of title compound, P(O)[NHC(O)C_6_H_3_(2,6-F_2_)][N(CH_3_)(CH_2_C_6_H_5_)]_2_. The phosphoryl and carbonyl groups are *anti* to each other and the phosphorus atom has a slightly distorted tetrahedral configuration (Fig. 1). The bond angles around the P atom are in the range of 107.11 (8)°-114.81 (8)°. The P1–N2 and P1–N3 bond lengths (1.6405 (14) Å and 1.6266 (16) Å) are shorter than the P1–N1 bond (1.6886 (15) Å). The environment of the nitrogen atoms is essentially planar; the angles C23–N3–P1, C16–N3–C23 and C16–N3–P1 are 117.53 (14)°, 116.19 (17)° and 126.13 (14)°, respectively (with average = 119.9°). A similar result was obtained for the bond angles around N2 atom (average = 119.1°). Furthermore, the angle C7–N1–P1 is 126.79 (13)°. The P═O bond length of 1.4796 (13) Å is standard for phosphoramidate compounds. The hydrogen atom of the C(═O)NHP(═O) group is involved in an intermolecular –P═O···H–N– hydrogen bond (see Table 1) to form a centrosymmetric dimeric aggregate.

### Experimental

The reaction of phosphorus pentachloride (3.478 g, 16.7 mmol) and 2,6-difluorobenzamide (2.624 g, 16.7 mmol) in dry CCl_4_ at 358 K (3 h) and then the treatment of formic acid (0.769 g, 16.7 mmol) at ice bath temperature leads to 2,6-F_2_—C_6_H_3_C(O)NHP(O)Cl_2_. To a solution of 2,6-F_2_—C_6_H_3_C(O)NHP(O)Cl_2_ (0.411 g, 1.5 mmol) in dry CHCl_3_, a solution of *N*-methylbenzylamine (0.727 g, 6 mmol) in dry CHCl_3_ was added dropwise at 273 K. After 4 h stirring, the solvent was evaporated in vacuum. The solid was washed with distilled water. Single crystals were obtained from a solution of the title compound in CH_3_OH and n-C_7_H_14_ (5:1) after slow evaporation at room temperature. IR (KBr, cm^-1^): 3446, 3042, 2867, 1690, 1607, 1465, 1369, 1210, 1150, 1019, 865, 823, 763, 725.

### Refinement

*APEX2* software was used for preliminary determination of the unit cell. Determination of integral intensities and unit cell refinement were performed using *SAINT* and data were corrected for absorption using *SADABS*. Structure was solved by direct methods and all non-hydrogen atoms were refined as anisotropic by Fourier full matrix least squares. Hydrogen H1A was found from a Fourier difference map and was allowed to refine and all other hydrogen atoms were placed in calculated positions with appropriate riding factors.

### Crystal data

**Table:**

C_23_H_24_F_2_N_3_O_2_P	*Z* = 2
*M**_r_* = 443.42	*F*(000) = 464
Triclinic, *P*1	*D*_x_ = 1.318 Mg m^−^^3^
*a* = 9.9370 (11) Å	Mo *K*α radiation, λ = 0.71073 Å
*b* = 11.1093 (15) Å	Cell parameters from 5531 reflections
*c* = 11.5902 (14) Å	θ = 2.4–27.8°
α = 89.101 (4)°	µ = 0.16 mm^−^^1^
β = 67.826 (4)°	*T* = 200 K
γ = 71.664 (4)°	BLOCK, colorless
*V* = 1116.9 (2) Å^3^	0.30 × 0.25 × 0.20 mm

### Data collection

**Table:**

Bruker SMART X2S benchtop CCD area-detector diffractometer	5173 independent reflections
Radiation source: fine-focus sealed tube	3851 reflections with *I* > 2σ(*I*)
curved silicon crystal	*R*_int_ = 0.036
phi and ω scans	θ_max_ = 27.8°, θ_min_ = 2.4°
Absorption correction: multi-scan *SADABS* (Bruker, 2005)	*h* = −13→13
*T*_min_ = 0.952, *T*_max_ = 0.968	*k* = −14→14
13602 measured reflections	*l* = −15→15

### Refinement

**Table:**

Refinement on *F*^2^	Primary atom site location: structure-invariant direct methods
Least-squares matrix: full	Secondary atom site location: difference Fourier map
*R*[*F*^2^ > 2σ(*F*^2^)] = 0.048	Hydrogen site location: inferred from neighbouring sites
*wR*(*F*^2^) = 0.126	H atoms treated by a mixture of independent and constrained refinement
*S* = 1.03	*w* = 1/[σ^2^(*F*_o_^2^) + (0.0603*P*)^2^ + 0.2218*P*] where *P* = (*F*_o_^2^ + 2*F*_c_^2^)/3
5173 reflections	(Δ/σ)_max_ = 0.001
286 parameters	Δρ_max_ = 0.37 e Å^−^^3^
0 restraints	Δρ_min_ = −0.32 e Å^−^^3^

### Special details

**Table:**

Geometry. All e.s.d.'s (except the e.s.d. in the dihedral angle between two l.s. planes) are estimated using the full covariance matrix. The cell e.s.d.'s are taken into account individually in the estimation of e.s.d.'s in distances, angles and torsion angles; correlations between e.s.d.'s in cell parameters are only used when they are defined by crystal symmetry. An approximate (isotropic) treatment of cell e.s.d.'s is used for estimating e.s.d.'s involving l.s. planes.
Refinement. Refinement of *F*^2^ against ALL reflections. The weighted *R*-factor *wR* and goodness of fit *S* are based on *F*^2^, conventional *R*-factors *R* are based on *F*, with *F* set to zero for negative *F*^2^. The threshold expression of *F*^2^ > σ(*F*^2^) is used only for calculating *R*-factors(gt) *etc*. and is not relevant to the choice of reflections for refinement. *R*-factors based on *F*^2^ are statistically about twice as large as those based on *F*, and *R*- factors based on ALL data will be even larger.

### Fractional atomic coordinates and isotropic or equivalent isotropic displacement parameters (Å^2^)

**Table:**

	*x*	*y*	*z*	*U*_iso_*/*U*_eq_	
P1	0.66485 (5)	0.42270 (4)	0.29906 (4)	0.02461 (13)	
F1	0.41237 (17)	0.18210 (16)	0.50507 (15)	0.0723 (5)	
F2	0.16095 (14)	0.43844 (12)	0.27352 (13)	0.0532 (4)	
O1	0.50833 (16)	0.27969 (15)	0.19272 (13)	0.0445 (4)	
O2	0.68882 (13)	0.46617 (12)	0.40757 (12)	0.0323 (3)	
N1	0.48905 (16)	0.40601 (15)	0.35526 (15)	0.0277 (3)	
N2	0.79630 (15)	0.28474 (13)	0.22927 (13)	0.0246 (3)	
N3	0.66719 (17)	0.52094 (15)	0.19333 (15)	0.0358 (4)	
C1	0.2810 (2)	0.2390 (2)	0.4858 (2)	0.0449 (5)	
C2	0.1464 (3)	0.2189 (3)	0.5632 (2)	0.0583 (7)	
H2A	0.1443	0.1678	0.6299	0.070*	
C3	0.0152 (3)	0.2755 (3)	0.5402 (2)	0.0573 (7)	
H3A	−0.0785	0.2634	0.5924	0.069*	
C4	0.0174 (2)	0.3490 (2)	0.4434 (2)	0.0485 (6)	
H4A	−0.0733	0.3880	0.4282	0.058*	
C5	0.1560 (2)	0.3646 (2)	0.36861 (19)	0.0370 (5)	
C6	0.2916 (2)	0.31110 (18)	0.38644 (17)	0.0315 (4)	
C7	0.4403 (2)	0.32953 (18)	0.30107 (17)	0.0309 (4)	
C8	0.86123 (19)	0.24379 (17)	0.09447 (16)	0.0280 (4)	
H8A	0.8268	0.3173	0.0509	0.034*	
H8B	0.8212	0.1771	0.0792	0.034*	
C9	1.03733 (19)	0.19106 (16)	0.03916 (16)	0.0250 (4)	
C10	1.1236 (2)	0.22336 (17)	0.09620 (17)	0.0296 (4)	
H10A	1.0727	0.2797	0.1720	0.035*	
C11	1.2842 (2)	0.17395 (19)	0.04339 (18)	0.0341 (4)	
H11A	1.3419	0.1964	0.0836	0.041*	
C12	1.3599 (2)	0.09219 (18)	−0.06744 (19)	0.0358 (5)	
H12A	1.4693	0.0584	−0.1033	0.043*	
C13	1.2756 (2)	0.06030 (19)	−0.12524 (19)	0.0393 (5)	
H13A	1.3270	0.0049	−0.2017	0.047*	
C14	1.1147 (2)	0.10908 (18)	−0.07202 (18)	0.0336 (4)	
H14A	1.0575	0.0859	−0.1123	0.040*	
C15	0.8149 (2)	0.18153 (18)	0.30902 (18)	0.0351 (4)	
H15A	0.9229	0.1261	0.2767	0.053*	
H15B	0.7497	0.1313	0.3084	0.053*	
H15C	0.7842	0.2184	0.3951	0.053*	
C16	0.5493 (2)	0.56887 (19)	0.14191 (18)	0.0382 (5)	
H16A	0.4917	0.5083	0.1535	0.046*	
H16B	0.6010	0.5705	0.0506	0.046*	
C17	0.4345 (2)	0.70157 (17)	0.20021 (17)	0.0290 (4)	
C18	0.3442 (2)	0.76890 (19)	0.13718 (19)	0.0360 (5)	
H18A	0.3538	0.7308	0.0604	0.043*	
C19	0.2409 (2)	0.8908 (2)	0.1858 (2)	0.0402 (5)	
H19A	0.1796	0.9355	0.1426	0.048*	
C20	0.2266 (2)	0.9478 (2)	0.2971 (2)	0.0414 (5)	
H20A	0.1568	1.0319	0.3297	0.050*	
C21	0.3144 (2)	0.88166 (19)	0.36020 (18)	0.0399 (5)	
H21A	0.3047	0.9202	0.4368	0.048*	
C22	0.4176 (2)	0.75831 (18)	0.31215 (18)	0.0344 (4)	
H22A	0.4767	0.7130	0.3569	0.041*	
C23	0.7939 (3)	0.5743 (2)	0.1510 (3)	0.0603 (7)	
H23A	0.8498	0.5537	0.0599	0.091*	
H23B	0.8646	0.5376	0.1925	0.091*	
H23C	0.7516	0.6673	0.1724	0.091*	
H1A	0.429 (2)	0.442 (2)	0.427 (2)	0.038 (6)*	

### Atomic displacement parameters (Å^2^)

**Table:**

	*U*^11^	*U*^22^	*U*^33^	*U*^12^	*U*^13^	*U*^23^
P1	0.0173 (2)	0.0247 (2)	0.0261 (2)	−0.00350 (17)	−0.00496 (17)	−0.00334 (17)
F1	0.0611 (9)	0.0976 (12)	0.0788 (11)	−0.0319 (9)	−0.0460 (8)	0.0389 (9)
F2	0.0449 (7)	0.0539 (8)	0.0632 (9)	−0.0087 (6)	−0.0301 (7)	0.0035 (7)
O1	0.0354 (8)	0.0614 (10)	0.0329 (8)	−0.0183 (7)	−0.0068 (6)	−0.0151 (7)
O2	0.0208 (6)	0.0398 (7)	0.0315 (7)	−0.0084 (5)	−0.0061 (5)	−0.0112 (6)
N1	0.0182 (7)	0.0342 (8)	0.0262 (8)	−0.0076 (6)	−0.0044 (6)	−0.0074 (7)
N2	0.0206 (7)	0.0242 (7)	0.0231 (7)	−0.0022 (6)	−0.0064 (6)	0.0009 (6)
N3	0.0259 (8)	0.0294 (8)	0.0446 (10)	−0.0035 (7)	−0.0107 (7)	0.0096 (7)
C1	0.0398 (12)	0.0603 (14)	0.0426 (12)	−0.0222 (11)	−0.0200 (10)	0.0059 (11)
C2	0.0607 (16)	0.0738 (18)	0.0467 (14)	−0.0402 (14)	−0.0133 (12)	0.0104 (13)
C3	0.0415 (13)	0.0695 (17)	0.0565 (15)	−0.0329 (13)	−0.0022 (11)	−0.0100 (13)
C4	0.0249 (10)	0.0540 (14)	0.0626 (15)	−0.0107 (10)	−0.0140 (10)	−0.0199 (12)
C5	0.0305 (10)	0.0391 (11)	0.0407 (11)	−0.0086 (8)	−0.0152 (9)	−0.0073 (9)
C6	0.0242 (9)	0.0382 (10)	0.0320 (10)	−0.0110 (8)	−0.0100 (8)	−0.0080 (8)
C7	0.0229 (9)	0.0361 (10)	0.0314 (10)	−0.0061 (8)	−0.0110 (8)	−0.0053 (8)
C8	0.0242 (9)	0.0297 (9)	0.0241 (9)	−0.0045 (7)	−0.0065 (7)	−0.0026 (7)
C9	0.0220 (8)	0.0247 (9)	0.0249 (9)	−0.0068 (7)	−0.0062 (7)	0.0030 (7)
C10	0.0291 (9)	0.0305 (9)	0.0268 (9)	−0.0079 (8)	−0.0100 (8)	0.0001 (7)
C11	0.0277 (10)	0.0394 (11)	0.0377 (11)	−0.0139 (8)	−0.0135 (8)	0.0078 (9)
C12	0.0202 (9)	0.0349 (10)	0.0431 (11)	−0.0073 (8)	−0.0042 (8)	0.0062 (9)
C13	0.0289 (10)	0.0364 (11)	0.0366 (11)	−0.0061 (8)	0.0008 (8)	−0.0093 (9)
C14	0.0275 (9)	0.0347 (10)	0.0341 (10)	−0.0113 (8)	−0.0063 (8)	−0.0046 (8)
C15	0.0328 (10)	0.0314 (10)	0.0344 (10)	−0.0050 (8)	−0.0106 (8)	0.0058 (8)
C16	0.0437 (11)	0.0322 (10)	0.0317 (10)	−0.0024 (9)	−0.0158 (9)	0.0019 (8)
C17	0.0267 (9)	0.0288 (9)	0.0299 (9)	−0.0078 (7)	−0.0107 (8)	0.0061 (8)
C18	0.0359 (10)	0.0403 (11)	0.0377 (11)	−0.0136 (9)	−0.0202 (9)	0.0070 (9)
C19	0.0299 (10)	0.0413 (12)	0.0511 (13)	−0.0066 (9)	−0.0222 (9)	0.0147 (10)
C20	0.0324 (10)	0.0326 (11)	0.0463 (12)	−0.0009 (8)	−0.0100 (9)	0.0057 (9)
C21	0.0426 (12)	0.0362 (11)	0.0322 (11)	−0.0068 (9)	−0.0103 (9)	0.0014 (9)
C22	0.0364 (10)	0.0304 (10)	0.0323 (10)	−0.0030 (8)	−0.0158 (8)	0.0060 (8)
C23	0.0362 (12)	0.0495 (14)	0.0853 (19)	−0.0157 (11)	−0.0129 (12)	0.0317 (13)

### Geometric parameters (Å, °)

**Table:**

P1—O2	1.4796 (13)	C10—H10A	0.9500
P1—N3	1.6266 (16)	C11—C12	1.385 (3)
P1—N2	1.6405 (14)	C11—H11A	0.9500
P1—N1	1.6886 (15)	C12—C13	1.377 (3)
F1—C1	1.360 (2)	C12—H12A	0.9500
F2—C5	1.359 (2)	C13—C14	1.397 (3)
O1—C7	1.219 (2)	C13—H13A	0.9500
N1—C7	1.362 (2)	C14—H14A	0.9500
N1—H1A	0.83 (2)	C15—H15A	0.9800
N2—C8	1.463 (2)	C15—H15B	0.9800
N2—C15	1.471 (2)	C15—H15C	0.9800
N3—C16	1.462 (2)	C16—C17	1.524 (3)
N3—C23	1.474 (3)	C16—H16A	0.9900
C1—C6	1.383 (3)	C16—H16B	0.9900
C1—C2	1.386 (3)	C17—C22	1.380 (3)
C2—C3	1.380 (4)	C17—C18	1.397 (3)
C2—H2A	0.9500	C18—C19	1.385 (3)
C3—C4	1.375 (3)	C18—H18A	0.9500
C3—H3A	0.9500	C19—C20	1.384 (3)
C4—C5	1.386 (3)	C19—H19A	0.9500
C4—H4A	0.9500	C20—C21	1.378 (3)
C5—C6	1.383 (3)	C20—H20A	0.9500
C6—C7	1.512 (3)	C21—C22	1.396 (3)
C8—C9	1.529 (2)	C21—H21A	0.9500
C8—H8A	0.9900	C22—H22A	0.9500
C8—H8B	0.9900	C23—H23A	0.9800
C9—C14	1.389 (2)	C23—H23B	0.9800
C9—C10	1.389 (2)	C23—H23C	0.9800
C10—C11	1.395 (2)		
			
O2—P1—N3	114.81 (8)	C12—C11—C10	120.19 (18)
O2—P1—N2	110.54 (7)	C12—C11—H11A	119.9
N3—P1—N2	107.66 (8)	C10—C11—H11A	119.9
O2—P1—N1	107.11 (8)	C13—C12—C11	119.55 (17)
N3—P1—N1	107.24 (8)	C13—C12—H12A	120.2
N2—P1—N1	109.34 (8)	C11—C12—H12A	120.2
C7—N1—P1	126.79 (13)	C12—C13—C14	120.31 (18)
C7—N1—H1A	116.4 (14)	C12—C13—H13A	119.8
P1—N1—H1A	116.5 (14)	C14—C13—H13A	119.8
C8—N2—C15	115.44 (14)	C9—C14—C13	120.73 (18)
C8—N2—P1	125.15 (12)	C9—C14—H14A	119.6
C15—N2—P1	116.63 (11)	C13—C14—H14A	119.6
C16—N3—C23	116.19 (17)	N2—C15—H15A	109.5
C16—N3—P1	126.13 (14)	N2—C15—H15B	109.5
C23—N3—P1	117.53 (14)	H15A—C15—H15B	109.5
F1—C1—C6	117.31 (18)	N2—C15—H15C	109.5
F1—C1—C2	119.0 (2)	H15A—C15—H15C	109.5
C6—C1—C2	123.7 (2)	H15B—C15—H15C	109.5
C3—C2—C1	117.9 (2)	N3—C16—C17	114.79 (16)
C3—C2—H2A	121.1	N3—C16—H16A	108.6
C1—C2—H2A	121.1	C17—C16—H16A	108.6
C4—C3—C2	121.4 (2)	N3—C16—H16B	108.6
C4—C3—H3A	119.3	C17—C16—H16B	108.6
C2—C3—H3A	119.3	H16A—C16—H16B	107.5
C3—C4—C5	118.0 (2)	C22—C17—C18	118.77 (17)
C3—C4—H4A	121.0	C22—C17—C16	122.46 (16)
C5—C4—H4A	121.0	C18—C17—C16	118.76 (17)
F2—C5—C6	117.05 (18)	C19—C18—C17	120.47 (19)
F2—C5—C4	119.21 (19)	C19—C18—H18A	119.8
C6—C5—C4	123.7 (2)	C17—C18—H18A	119.8
C5—C6—C1	115.30 (18)	C20—C19—C18	120.37 (18)
C5—C6—C7	121.65 (18)	C20—C19—H19A	119.8
C1—C6—C7	123.04 (17)	C18—C19—H19A	119.8
O1—C7—N1	123.93 (17)	C21—C20—C19	119.48 (19)
O1—C7—C6	121.61 (16)	C21—C20—H20A	120.3
N1—C7—C6	114.46 (15)	C19—C20—H20A	120.3
N2—C8—C9	112.58 (14)	C20—C21—C22	120.33 (19)
N2—C8—H8A	109.1	C20—C21—H21A	119.8
C9—C8—H8A	109.1	C22—C21—H21A	119.8
N2—C8—H8B	109.1	C17—C22—C21	120.56 (18)
C9—C8—H8B	109.1	C17—C22—H22A	119.7
H8A—C8—H8B	107.8	C21—C22—H22A	119.7
C14—C9—C10	118.49 (16)	N3—C23—H23A	109.5
C14—C9—C8	119.50 (15)	N3—C23—H23B	109.5
C10—C9—C8	122.01 (15)	H23A—C23—H23B	109.5
C9—C10—C11	120.73 (17)	N3—C23—H23C	109.5
C9—C10—H10A	119.6	H23A—C23—H23C	109.5
C11—C10—H10A	119.6	H23B—C23—H23C	109.5

### Hydrogen-bond geometry (Å, °)

**Table:**

*D*—H···*A*	*D*—H	H···*A*	*D*···*A*	*D*—H···*A*
N1—H1A···O2^i^	0.83 (2)	1.92 (2)	2.752 (2)	173 (2)

Symmetry codes: (i) −*x*+1, −*y*+1, −*z*+1.
